# “Severe Aortic Regurgitation as the Initial Manifestation of Takayasu Arteritis in a Young Woman: A Case Report”

**DOI:** 10.1155/carm/5542781

**Published:** 2026-09-15

**Authors:** Amir Heidari, Azar Nejati, S. Enssieh Hashemi, Mohammad Javad Bahadori, Golnaz Houshmand, Amin Fath Tabar

**Affiliations:** ^1^ Clinical Research Development Center, Shahid Beheshti University of Medical Sciences Imam Hossein Educational Hospital, Tehran, Iran; ^2^ Student Research Committee, Shahid Beheshti University of Medical Sciences School of Medicine, Tehran, Iran; ^3^ Department of Neurology, Iran University of Medical Sciences, Tehran, Iran, iums.ac.ir; ^4^ Rajaie Cardiovascular Medical and Research Institute, Tehran, Iran; ^5^ Cardiovascular Imaging Research Center, Rajaie Cardiovascular Institute, Tehran, Iran

**Keywords:** aortic regurgitation, case report, large-vessel vasculitis, Takayasu arteritis, valvular dysfunction

## Abstract

Takayasu arteritis (TAK) is a rare large‐vessel vasculitis primarily affecting young women, characterized by idiopathic inflammation of the aorta and its major branches. While aortic regurgitation (AR) is a recognized complication, it typically occurs in advanced disease stages. Isolated severe AR as the initial manifestation is exceptionally uncommon and poses diagnostic challenges. We present a 39‐year‐old woman with no prior medical history who presented with progressive exertional dyspnea (NYHA Class II) and exertional chest pain. Physical examination revealed absent upper limb pulses, unmeasurable brachial blood pressure, and a diastolic murmur. Transthoracic echocardiography demonstrated severe AR with left ventricular dysfunction (EF 48%). CT and MR angiography confirmed circumferential aortic wall thickening, subclavian artery occlusions, and ostial right coronary artery stenosis. Elevated inflammatory markers (ESR and CRP) and vascular imaging findings established the diagnosis of TAK based on the 1996 Modified Ishikawa Criteria. Initial treatment included high‐dose glucocorticoids and methotrexate for disease control. Due to persistent severe AR, the patient underwent successful mechanical aortic valve replacement. Postoperative recovery was uncomplicated, with significant clinical improvement at 1‐year follow‐up and no disease recurrence. This case illustrates that severe AR can rarely serve as the initial manifestation of TAK, emphasizing the importance of considering large‐vessel vasculitis in young patients with unexplained valvulopathy and systemic inflammation. Early diagnosis, combined medical‐surgical management, and careful long‐term monitoring are essential for optimizing outcomes in this high‐risk population.

## 1. Introduction

Takayasu arteritis (TAK) is a rare large‐vessel vasculitis, with an estimated incidence of one to three cases per million individuals. It is characterized by chronic idiopathic inflammation of large vessels, stemming from immune‐mediated processes, vascular remodeling, and genetic factors. This condition is more prevalent among women in their twenties to thirties [1, 2]. TAK primarily affects the aorta and its major branches leading to complications such as arterial stenosis, aneurysms, and valvular dysfunction. The clinical presentation of TAK is highly variable. In a recent descriptive study of 43 case reports, Alnabwani et al. found that constitutional symptoms such as fever (20.93%) and nonspecific complaints including chest pain (13.95%), claudication (13.95%), and headache (13.95%) were the most frequent initial manifestations, while valvular involvement was uncommon, with AR detected on echocardiography in only 4.65% of cases [3]. Among these, AR is a high‐risk complication associated with increased mortality. While AR typically occurs in advanced stages of TAK, isolated severe AR as the initial manifestation is exceedingly rare. Surgical intervention for this complication is particularly challenging, and due to the inflammatory nature of the vascular walls, any surgical procedures should ideally be postponed until the disease is under control [4].

Additionally, involvement of the coronary arteries can occur in seven to 9% of TAK cases, underscoring the importance of timely diagnosis and treatment [2]. Patients with TAK may present with nonspecific symptoms, such as fever, fatigue, myalgia, weight loss, night sweats, and arthralgia in the early stages. Consequently, they may remain undiagnosed for months or even years, or their symptoms may be attributed to other causes [1, 2]. The chronic nature of the disease and the need for prolonged immunosuppressive therapy pose challenges to the quality of life and survival of affected patients, further emphasizing the necessity for accurate diagnosis and appropriate management.

In this report, we present the case of a young female patient who initially manifested with exertional dyspnea. Investigations revealed severe AR and the absence of pulse, along with unmeasurable blood pressure in the upper limbs, ultimately leading to a diagnosis of TAK. This case highlights an unusual presentation where severe AR, rather than classic limb ischemia or vascular bruits, was the primary symptom leading to diagnosis. The significance of this case lies in the fact that AR typically occurs at later stages of TAK; however, our patient presented early with exertional dyspnea resulting from severe AR. This highlights the importance of recognizing less common manifestations of the disease for accurate diagnosis and treatment.

## 2. Case Presentation

A 39‐year‐old woman with no prior medical history presented to our clinic with complaints of fatigue and dyspnea on exertion. Her dyspnea had begun 3 years earlier and had worsened over the past month, reaching New York Heart Association (NYHA) Class II. She also reported experiencing severe chest pain during physical activity over the past 2 weeks. She did not report symptoms suggestive of upper limb ischemia, such as arm claudication or ischemic limb pain. She had no previous pregnancies and was nulligravid.

Physical examination revealed absent pulses in both upper limbs and nonrecordable brachial blood pressure bilaterally. Blood pressure was measured in the lower limbs, revealing hypertension (average reading: 150/90 mmHg), later confirmed via arterial catheterization during coronary angiography. A diastolic murmur was detected at the left sternal border. During the abdominal examination, a bruit was auscultated over the right renal artery.

**FIGURE 1:**
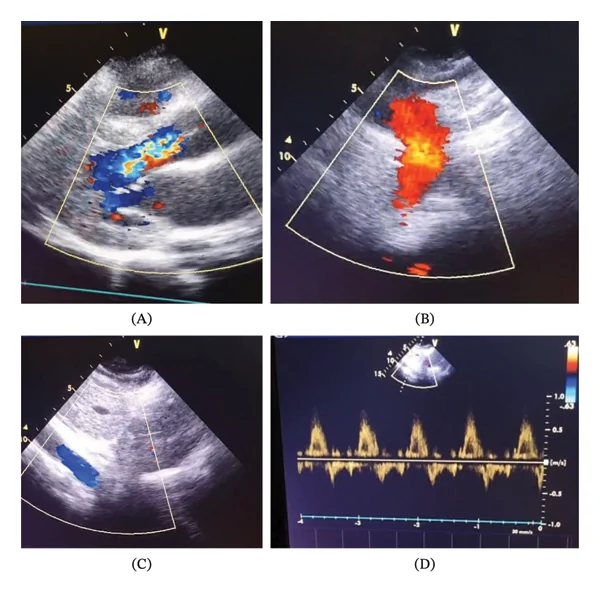
(A) Parasternal long‐axis view echocardiogram showing severe AI on color Doppler. (B) Holodiastolic flow reversal in descending aorta in suprasternal notch view on color Doppler and (C, D) holodiastolic flow reversal in abdominal aorta in subcostal view on color Doppler (C) and pulsed‐wave Doppler (white arrow) (D) consistent with severe aortic regurgitation.

**FIGURE 2:**
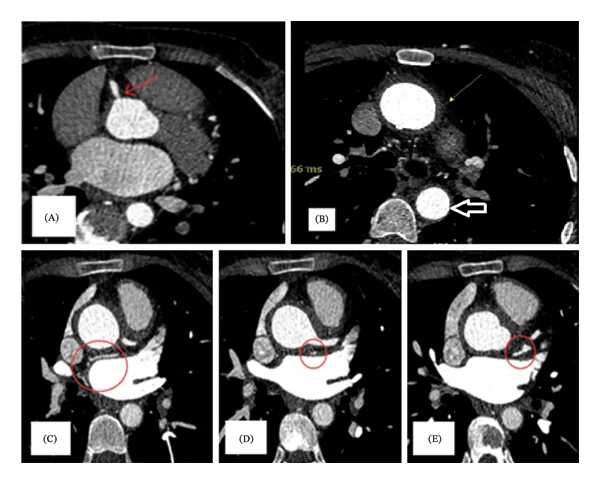
(A) Right coronary artery ostial narrowing in CT angiography, (B) CTA in axial view showing increased wall thickness in ascending aorta (thin arrow) and descending aorta (thick white arrow), (C–E) SA node artery fistula, branching from LCX in CT angiography.

An emergency electrocardiogram showed no abnormalities. The patient underwent a transthoracic echocardiography, which, using the Simpson method revealed an ejection fraction (EF) of 48% and severe aortic insufficiency (AI) with retrograde flow in the abdominal aorta (Figure 1). Severe aortic insufficiency was clearly demonstrated by holodiastolic flow reversal in the abdominal aorta (Supporting Video S1) and descending aorta (Supporting Video S2), as well as in the apical 5‐chamber view (Supporting Video S3). Additional detailed echocardiographic views, including parasternal long‐axis views, further corroborated these findings (Supporting Videos S4 and S5). The mitral valve was also assessed, revealing mitral regurgitation (Supporting Video S6), and additional views of the mitral valve leaflets were obtained (Supporting Video S7). According to the patient’s symptoms of dyspnea and chest pain on exertion, low EF, and evidence of severe AR in echocardiography, an aortic and coronary CT angiography was performed. CT angiography revealed moderate ostial stenosis, and wall thickening of the right coronary artery (RCA) (Figure 2A), and circumferential wall thickening of the aorta (from root to abdominal aorta) (Figure 2B) consistent with vasculitis. No evidence of aortic aneurysm or dissection was observed. Incidentally, we also identified a sinoatrial (SA) nodal artery arising from the left circumflex artery (LCX) and supplying a bronchial artery (Figure 2C). Subsequent invasive coronary angiography with fractional flow reserve (FFR) assessment of the RCA ostial lesion was negative, indicating no hemodynamically significant stenosis. Laboratory data indicated an elevated erythrocyte sedimentation rate (ESR) of 84 mm/h, raised C‐reactive protein (CRP) of 35 mg/L, serum creatinine of 1.0 mg/dL, potassium of 4.0 mEq/L, and sodium of 138 mEq/L.

Inflammatory markers were first measured during the current presentation and had not been assessed previously, as the patient’s 3‐year history of dyspnea was attributed to noncardiac causes at prior evaluations. No echocardiography or vascular imaging had been performed before this admission. Although the prolonged symptom duration implies longstanding vascular inflammation, severe AR—rather than classic ischemic symptoms such as arm claudication or syncope—was the dominant clinical manifestation that ultimately led to the diagnosis.

**FIGURE 3:**
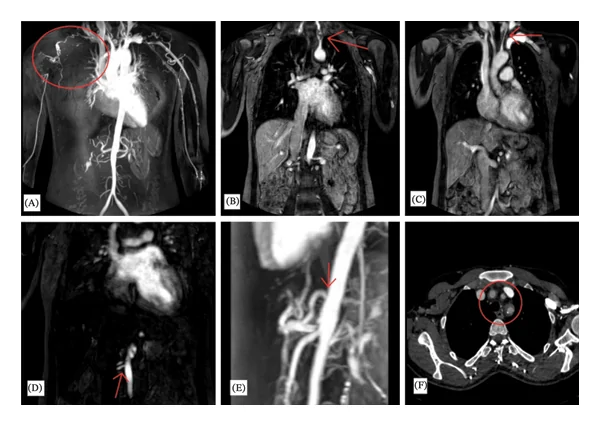
MR angiography: (A) right subclavian artery cutoff, (B) left subclavian artery cutoff, (C) left common carotid artery narrowing, (D) right renal artery ostial narrowing, (E) celiac artery ostial narrowing, (F) thickening of the wall of the aortic branches.

**FIGURE 4:**
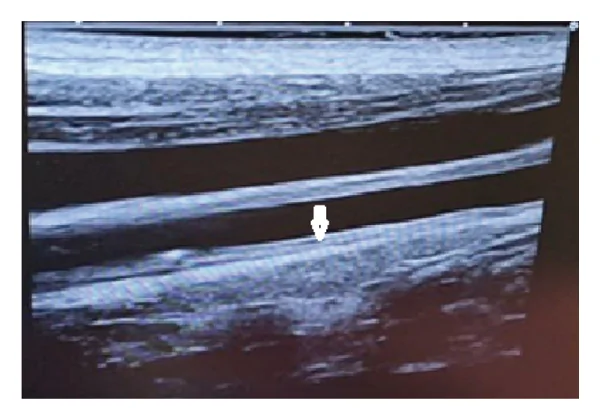
Carotid artery sonography showing thickening of the carotid artery wall, increased carotid intima‐media thickness consistent with macaroni sign in Takayasu arteritis (white arrow).

Given her age, constitutional symptoms, pulseless upper extremities, elevated inflammatory markers such as ESR and CRP, circumferential wall thickening of the aorta, involvement of the coronary artery ostium, and increased thickness of the RCA wall, TAK was considered the primary differential diagnosis. The patient underwent MR angiography of the aorta, which confirmed increased wall thickness of the aortic root, ascending aorta, aortic arch, descending aorta, and abdominal aorta. Both subclavian arteries showed near‐total occlusion and were cut off in proximal parts, along with severe ostial stenosis at the celiac trunk and the right renal artery (Figure 3). Left common carotid artery was narrow with wall thickening at the proximal part and carotid Doppler ultrasound revealed increased wall thickness in common carotid arteries, consistent with the “macaroni sign” which is diffuse circumferential wall thickening (Figure 4, Supporting video S8). The finding of the right renal artery ostial stenosis on MRA (Figure 3D) provided an anatomical explanation for the patient’s hypertension and the right renal bruit auscultated on physical examination. DSA was not performed, as the diagnosis was clearly established on noninvasive CT and MR angiography. Given the conclusive imaging findings and the absence of an indication for endovascular intervention, the additional risks of an invasive angiographic procedure were considered unnecessary in this young patient.

Differential diagnoses considered included giant cell arteritis, which was excluded based on age (GCA rarely occurs under 50 years) [5], and polyarteritis nodosa, which predominantly affects medium‐sized muscular arteries [6] and is not typically associated with large‐vessel involvement, aortic wall thickening, or AR, which were central to our patient’s presentation. Other causes of AR such as bicuspid aortic valve, infective endocarditis, connective tissue disorders, and aortic dissection were ruled out by echocardiography and clinical evaluation. The absence of an intimal flap or false lumen on echocardiography and cross‐sectional imaging excluded aortic dissection.

**FIGURE 5:**
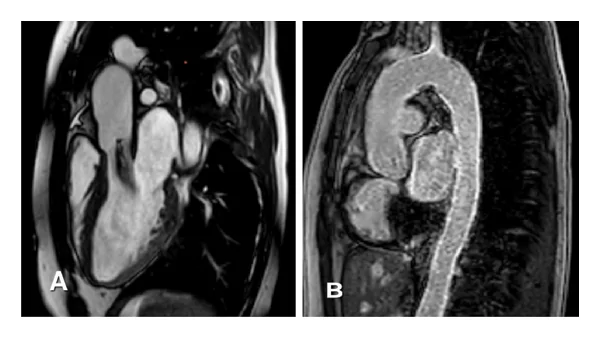
Cardiac magnetic resonance imaging: (A) three‐chamber balanced steady‐state free precession (SSFP) in diastole showing severe aortic regurgitation and (B) late gadolinium enhancement (LGE) image acquired in the candy cane view reveals diffuse enhancement of the thoracic aortic wall, suggesting active inflammation.

According to the 1996 Modified Ishikawa Criteria, the diagnosis of TAK was established by the presence of three major criteria (bilateral mid‐subclavian artery occlusive lesions on MRA and characteristic signs and symptoms lasting ≥ 1 month including exertional dyspnea and absent upper limb pulses) and four minor criteria: elevated ESR, hypertension, severe AR, and coronary artery involvement (RCA ostial stenosis). Additional findings, including proximal left common carotid artery narrowing and renal artery stenosis, provided further supportive evidence [7]. The patient’s cardiac MRI also revealed severe AR, along with late gadolinium enhancement in the aorta, which may indicate aortitis with acute inflammation (Figure 5).

Initially, the patient was treated with oral prednisolone regimen of 60 mg/day. Methotrexate (MTX) 10 mg weekly was added to taper the prednisolone dose and to achieve the glucocorticoid plus nonglucocorticoid immunosuppressant combination therapy. After 4 months of immunosuppressive therapy, despite a decline in ESR from 84 mm/hour to 17 mm/hour, the symptoms of severe aortic insufficiency persisted and the severity of AR remained unchanged. Therefore, the patient underwent surgical aortic valve replacement (AVR) with a mechanical valve. After a few days of recovery, she was discharged. Her dyspnea and other symptoms significantly improved during follow‐up visits and had no disease recurrence during the 1 year follow‐up.

## 3. Discussion

TAK is a form of large‐vessel vasculitis characterized by granulomatous inflammatory lesions affecting large vessels, including the aorta and its major branches that can also cause coronary artery stenosis and valvular damage, especially in the aortic valve. These damages, respective of the affected vessel, can cause complications such as acute coronary syndrome, cardiomyopathy, cerebrovascular accident, limb claudication, and mesenteric ischemia [8].

TAK is a triphasic disease. The first stage is systemic inflammation and prestenotic disease (prepulseless phase), which progresses to the second stage, which is a stenotic or aneurysmal injury with or without pain (pulseless phase). Finally, it goes through the third stage, burnt‐out fibrotic disease (fibrotic phase) [1, 9].

Various studies have reported the incidence of valvular involvement in TAK patients to be around 20%–60%, with aortic insufficiency being the most common form of involvement. The ascending aorta vessel wall inflammation can lead to fibrosis and dilation in the aortic root, causing aortic insufficiency [10].

Considering that valvular involvement can be associated with inflammatory processes, a study showed that disease activity parameters such as CRP, Kerr score, Indian Takayasu Arteritis Activity Score (ITAS), and ITAS‐a are higher when valvular involvement is present [10].

Aortic insufficiency in TAK does not usually become severe enough to require valve replacement and happens in later stages as a result of aortic root dilation [11]. While previous literature emphasizes that TAK should be considered in young women presenting primarily with angina and coronary ostial stenosis with AR being a serious downstream complication [11], in our patient, isolated severe AR was the primary and initial manifestation of the disease, rather than a late‐stage complication. Severe AR as the initial manifestation of TAK has been reported only sporadically in the literature. Furthermore, unlike classic presentations, our patient lacked aortic root dilation. Murayama et al. similarly described a patient in whom severe AR leading to heart failure was the presenting feature of newly diagnosed TAK; however, in their case, AR was secondary to aortic root dilation, and the patient exhibited the rare phenomenon of diastolic aortic valve opening—neither of which was observed in our patient [12]. Boparai et al. reported a case of TAK presenting with severe AR, but their patient had additional prominent features including a large pericardial effusion, pulmonary artery compression, and bounding pulses, whereas in our case, severe AR was the sole dominant clinical manifestation [13]. Hoshino et al. described severe AR due to TAK where diagnosis was delayed by concomitant osteomyelitis; in contrast, our patient had no such complicating comorbidity, yet the diagnosis was similarly delayed due to the absence of typical ischemic or pulseless symptoms [14]. Collectively, these comparisons demonstrate that our case is distinct in presenting with isolated severe AR—without aortic root dilation, without pericardial or pulmonary involvement, and without confounding comorbidities—as the sole clinical feature prompting the diagnostic workup for TAK.

### 3.1. Clinical Presentation and Diagnosis

In our patient, the clinical manifestations—including exertional dyspnea, exertional chest pain, the absence of palpable upper limb pulses, hypertension (>a0140/90 mmHg), severe AR, thinning of the coronary artery ostium, and increased wall thickness of the aorta and its major branches on imaging—strongly supported the diagnosis of TAK according to the 1996 Modified Ishikawa’s Criteria [7].

### 3.2. Treatment Strategy

Corticosteroids are the mainstay of treatment for TAK. Glucocorticoid‐sparing immunosuppressive agents should be used as adjunctive therapy in combination with glucocorticoids for all patients in the active phase. Biological agents may be considered in refractory or relapsing cases [4]. In our patient, treatment was initiated with high‐dose prednisolone and MTX. Regarding the potential use of biologic therapy, Interleukin‐6 (IL‐6) receptor inhibition with tocilizumab was considered but not administered. The patient achieved adequate disease control with glucocorticoids and MTX, as evidenced by a marked decline in ESR from 84 to 17 mm/h over four months of therapy. According to the 2018 EULAR guideline, biologic agents are recommended for patients with refractory or relapsing disease despite conventional immunosuppressive therapy [4]. In this patient, remission was successfully achieved with first‐line therapy, obviating the need for biologic escalation.

In our patient, initial treatment consisted of glucocorticoids combined with nonglucocorticoid immunosuppressive therapy (MTX), which effectively alleviated symptoms. However, due to persistent severe aortic insufficiency, the patient underwent surgical AVR to correct vascular abnormalities. At follow‐up, the patient showed improvement in dyspnea and aortic insufficiency symptoms, with no complications observed at the 1‐year mark.

Regarding surgical approach, combined aortic valve and root replacement (CAVRR) is more complex than isolated AVR but may offer better long‐term outcomes in terms of late postoperative cardiac events [15].

### 3.3. Surgical Challenges in TAK

In TAK, fragile and fibrotic tissues pose significant surgical challenges. Inflamed vessels are prone to rupture, which can lead to severe intra‐ or postoperative complications. Additionally, impaired healing due to underlying inflammation increases the risk of postoperative infections, poor wound healing, or further arterial damage.

Notable causes of postoperative mortality, morbidity, and reoperation following surgical management of AR in TAK—even with anti‐inflammatory therapy—include the following:

Prosthetic valve/graft dehiscence, pseudoaneurysm formation, periprosthetic leaks, detachment of prosthetic valves, and aneurysm formation at anastomotic sites [16].

Prognosis is better in patients who achieve disease remission with adequate immunosuppressive therapy prior to surgery [16]. However, life‐ or organ‐threatening manifestations (e.g., stroke, myocardial infarction, and limb ischemia) may necessitate emergency surgery even during the active phase [17–19]. High‐dose glucocorticoids should be administered preoperatively in such cases [20].

### 3.4. Coronary and Renal Involvement

In TAK patients with angina, coronary artery involvement—particularly in young women—must be considered. PCI carries a high risk of in‐stent restenosis in patients not on corticosteroids, making CABG the preferred option for symptomatic coronary disease refractory to medical therapy [16].

Our patient had ostial narrowing of the RCA on CT angiography but with nonsignificant stenosis (according to negative FFR), obviating the need for intervention.

For renovascular hypertension and renal artery stenosis, medical therapy (antihypertensive and immunosuppressant in active disease) is preferred over surgical intervention. In our patient, renal artery stenosis and hypertension were controlled medically. Catheter‐based or surgical intervention (e.g., stenting) may be considered in cases of refractory hypertension or worsening renal function [20].

In asymptomatic patients with cranial/cervical artery stenosis, medical management is favored over surgical intervention. Our patient had diffuse carotid wall thickening without cerebrovascular symptoms or significant stenosis.

Routine antiplatelet/anticoagulant therapy is not recommended for large‐vessel vasculitis unless indicated for comorbid conditions (e.g., CAD or cerebrovascular disease) [4]. In our case, low‐dose aspirin was not initiated despite coronary ostial involvement, as the patient already required lifelong warfarin for mechanical valve prosthesis, and the addition of aspirin to ongoing prednisolone therapy was considered to pose an unacceptably high bleeding risk. The patient was also started on atorvastatin for cardiovascular protection, given the coronary ostial involvement.

### 3.5. Monitoring and Long‐Term Management

Scheduled noninvasive imaging alongside routine clinical assessments is recommended in TAK, as vascular progression may occur despite clinical quiescence. The optimal imaging interval remains unclear but may range from 3 to 6 months or longer [20].

This case illustrates that severe AR—typically a late complication of TAK—can rarely serve as the initial manifestation, underscoring the importance of considering large‐vessel vasculitis in young patients with unexplained valvulopathy and systemic inflammation. The 3‐year diagnostic delay in this case likely reflects the rarity of TAK, the nonspecific nature of the patient’s initial symptom (exertional dyspnea), and the underrecognition of pulse deficits on a routine physical examination prior to her presentation at our center.

## Funding

No funding was received for this manuscript.

## Disclosure

The authors take full responsibility for the accuracy and integrity of all scientific content presented in this report.

## Ethics Statement

The ethics committee of Shahid Beheshti University of Medical Sciences has approved the conduct of this study (Ethics code: IR.SBMU.RETECH.REC.1403.484). The ethics committee certificate is available at https://ethics.research.ac.ir/ProposalCertificateEn.php?id=510366%26Print=true%26NoPrintHeader=true%26NoPrintFooter=true%26NoPrintPageBorder=true%26LetterPrint=true.

## Consent

The patient gave written informed consent for the publication of this case report. All the authors have approved a copy of the consent form, which is available for review by the editor of this journal.

## Conflicts of Interest

The authors declare no conflicts of interest.

## Supporting Information

Additional supporting information can be found online in the Supporting Information section.

## Supporting information


**Supporting Information 1** S1: Flow reversal in abdominal aorta.

**Supporting Information 2** S2: Flow reversal in thoracic descending aorta.

**Supporting Information 3** S3: Severe AI in 5CB: Apical 5 chamber view in echocardiography showing aortic insufficiency in the left ventricular outflow tract.

**Supporting Information 4** S4: Plax: Parasternal long axis view in echocardiography.

**Supporting Information 5** S5: Plax view: Parasternal long axis view in echocardiography.

**Supporting Information 6** S6: MR in 4CB view: Mitral regurgitation shown in 4‐chamber view.

**Supporting Information 7** S7: 4CB: 4‐chamber view in echocardiography showing rheumatic disease affecting anterior and posterior mitral valve leaflet.

**Supporting Information 8** S8: Carotid ultrasound: Inflammation and thickening of carotid artery wall in carotid ultrasound.

## Data Availability

The datasets used during the current study are available from the corresponding author on reasonable request.
